# Decoding BCL6 Inhibitors: Computational Insights into the Impact of Water Networks on Potency

**DOI:** 10.1021/acs.jcim.5c01188

**Published:** 2025-08-28

**Authors:** Daniella E. Hares, Andrea Scarpino, Michael S. Bodnarchuk, Swen Hoelder

**Affiliations:** † Centre for Cancer Drug Discovery, 5053 The Institute of Cancer Research, London SM2 5NG, U.K.; ‡ Oncology R&D, AstraZeneca, Cambridge Biomedical Campus, Cambridge CB2 0AA, U.K.

## Abstract

Water molecules in the binding site can have a critical role in small molecule binding to proteins and are an important consideration in structure-based drug design. Water networks have additional complexity as displacing one water molecule has subsequent effects on the remaining network. Modification of a lead compound that disrupts a water network can have beneficial or detrimental impacts on potency and this outcome is impossible to determine experimentally without time-consuming synthesis of the new compound. Computational methods are ideally suited to study the interplay between ligand optimization and water displacement by predicting the effect of structural changes on both the activity of the compound and the stability of neighboring water molecules. We used Grand Canonical Monte Carlo (GCMC) simulations and alchemical free energy calculations to retrospectively study a series of B-cell Lymphoma 6 (BCL6) inhibitors that sequentially displaced water molecules from a network. The methods were used to rationalize the structure–activity relationship of the compounds by quantifying the individual contributions to the binding affinity from the changes in the water network and new interactions with the protein. GCMC simulations are well-suited for studying water networks in the binding site and were able to reproduce 94% of the experimentally observed water sites from the crystal structures in a subpocket of BCL6. Using the BCL6 project as an example, we show the power of these computational methods to study water networks and how they can provide insights that are able to guide drug discovery projects.

## Graphical abstract

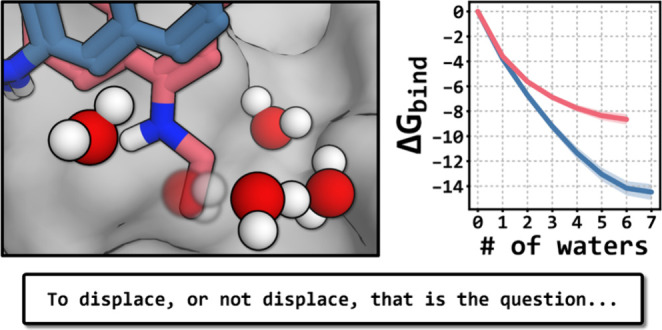

## Introduction

Water molecules in the binding site can have various effects on small molecule binding to proteins, such as influencing potency and selectivity, or stabilizing a particular orientation.^1–4^ These protein-bound water molecules often have limited motion in comparison to bulk water, with an entropic cost estimated to be up to 2 kcal mol^–1^.^5^ Their displacement can improve the binding affinity of small molecules, but usually the penalty of breaking the hydrogen bonds between the water molecules needs to be recovered through new interactions with the protein or remaining water molecules.^6,7^

It is challenging to experimentally determine the contribution of solvent effects toward changes in binding affinity. Therefore, a variety of computational methods have been developed to predict these thermodynamic values for the water molecules at the protein–ligand interface.^8^ Furthermore, these methods predict whether a change to a water network in the binding site will have a beneficial, neutral or detrimental effect on the overall affinity when modifying the lead structure. This can be powerful to identify opportunities to displace or interact with water molecules and retrospectively interpreting unexpected trends in structure–activity relationships.^9–12^

When a water molecule accepts a hydrogen bond, its electrons are redistributed to encourage the further donation of another hydrogen bond.^13^ This results in cooperativity of a water network in terms of hydrogen bond formation, where the total interaction strength of the network is greater than the sum of the individual interactions between the water molecules. The importance of considering water networks when growing into solvent-filled pockets has been recognized for drug design.^14–16^ Samways et al.^15^ performed a computational study of 108 proteins complexed with FDA-approved drugs and found over half of the structures were predicted to have a “ligand-contacting water network”. Ligand modifications that displace a water molecule could destabilize the remaining network and result in a detrimental loss in potency. In particular, Darby et al.^16^ found that displacing one water molecule from a network caused a 1400-fold decrease in ligand affinity despite the same binding mode. The drop in affinity was a result of a single residue mutation from alanine to asparagine that displaced a single water molecule and perturbed the remaining water network. This highlights that cooperative effects between water molecules are crucial factors to consider in structure-based drug design.

Unfortunately, computational solvent analysis methods often do not explicitly calculate interactions between water molecules, only considering the interactions of individual water molecules with the protein–ligand complex without capturing cooperative effects. One example is SZMAP,^17^ which can predict the relative free energy of a water molecule compared to an uncharged probe. These predicted values were shown by Bayden et al.^18^ to correlate with absolute binding free energies calculated using the rigorous double decoupling method. However, the correlation improved when water sites that made two or more hydrogen bonds to other water molecules were filtered out, with the R^2^ increasing from 0.43 to 0.66, implying that water networks may need to be considered in the modeling of these sites. Another computational method used for solvent analysis is 3D Reference Interaction Site Model (3D-RISM),^19^ which is derived from statistical mechanics and calculates the free energies at grid points around a rigid solute. 3D-RISM accounts for correlation effects, but its results are based on approximate distribution functions,^20^ which reduces its accuracy compared to more rigorous methods such as Monte Carlo simulations.

Grand Canonical Monte Carlo (GCMC)^21,22^ is a computational technique that implicitly accounts for the correlation between water molecules in a network. Similar to Molecular Dynamics (MD), GCMC can be used to explore the conformations of a system but uses random changes to the system configuration instead of showing how the system evolves over time. This means that GCMC simulations have the benefit over MD simulations that they can overcome local minima in the conformational landscape. Additionally, the number of water molecules in the system can vary during a GCMC simulation as water molecules can be inserted and deleted from a defined pocket in the protein. This is based on Metropolis Monte Carlo^23^ and the probability of the insertion or deletion is based on the applied chemical potential (B value).^24^ GCMC was used to predict the water locations in over 100 proteins complexed with FDA-approved drugs and identified 81.4% of the nonbulk water sites within 1.4 Å of the crystallographic position.^15^ GCMC simulations can be a complementary method to X-ray crystallography at predicting the location of water molecules in the protein:^15^ they are faster and less-resource intensive, and often can identify more disordered water sites than would be resolved experimentally. Therefore, GCMC predictions can be used to identify likely water sites when docking into crystal structures of a protein with similar ligands and there is no experimental data available. It is worth noting that GCMC is more reliable at predicting crystallographic water sites that have more hydrogen bonds to the protein or ligand compared to more disordered water sites that only make one hydrogen bond.^15^

Additionally, GCMC simulations can be used to predict the binding free energy (Δ*G*_bind_) of water molecules compared to bulk solvent, which is a straightforward and widely used measure of stability. For example, GCMC was used to rationalize the selectivity of a c-KIT inhibitor over KDR.^4^ The simulations identified water networks that were not visible in the X-ray crystal structures and predicted the water network in c-KIT to be 3.3 kcal mol^–1^ more stable than the network in KDR, helping to explain the selectivity profile.

These case studies illustrate the power of GCMC to study water molecules at the protein–ligand interface, but this method is underutilized in drug discovery possibly due to lack of general awareness and its absence from commercial software packages. To further illustrate the benefits of GCMC for drug design, additional examples are needed where the ligand modification perturbs a larger network of water. For instance, inhibitors of B-cell lymphoma 6 (BCL6) bind at the groove formed between the two chains of the homodimer and this binding site has an adjacent subpocket that contains a water network, shown in Figure 1a. Lloyd et al.^11^ designed a series of inhibitors that grow into this subpocket and solved high-resolution X-ray crystal structures of several compounds, providing the ideal system for computational analysis and molecular simulation.

**Figure 1:**
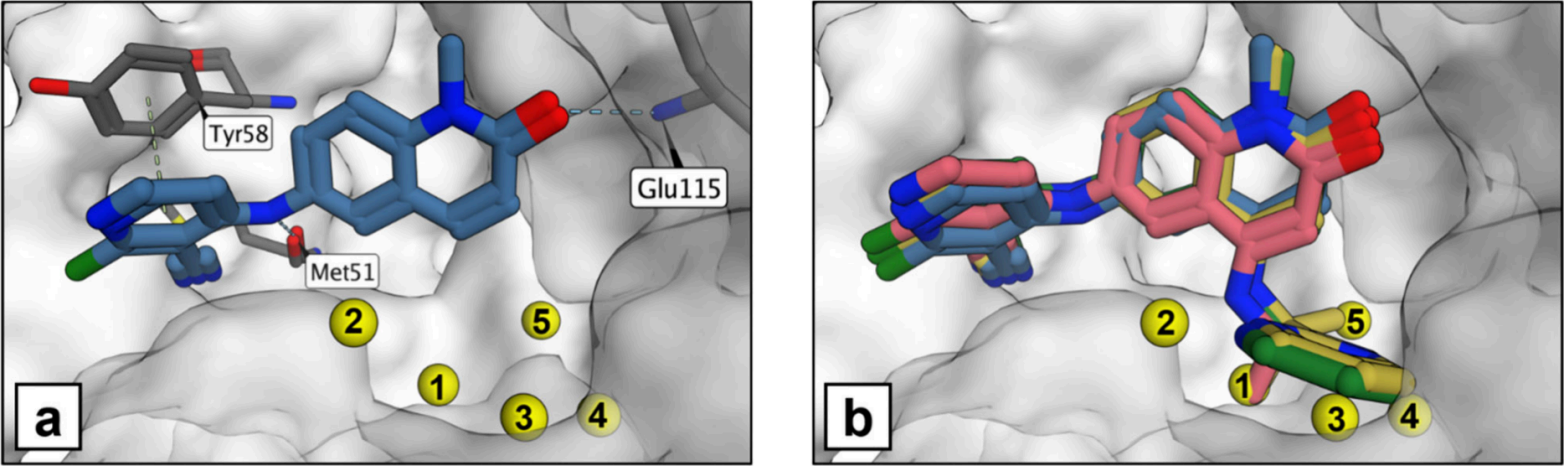
(a) Crystal structure of compound **1** (PDB ID: 7OKE) showing the key protein residues in gray, compound **1** in blue and the location of the water network in yellow. (b) Crystal structure of compound **1** in blue overlaid with compounds **2**, **3**, and **4** (PDB IDs: 7OKH, 7OKL, 7OKM - ligand only), shown in pink, green, and yellow, respectively.

Our aim was to test the ability of GCMC to rationalize potency changes resulting from the perturbation of this water network in BCL6. Therefore, we selected four compounds with different sized substituents that sequentially displace up to three water molecules from the network, achieving a 50-fold improvement in potency. The key details for these compounds are summarized in Table 1. The compounds have a common scaffold that makes several key interactions with the protein as shown in Figure 1a, including hydrogen bonds to Met51 and Glu115. The pyridyl group forms a stacking interaction with the Tyr58 side chain. The subpocket within the protein adjacent to the quinolinone group of compound **1** contains a network of five water molecules. Figure 1b shows an overlay with compounds **2**–**4**, which have substituents extending from the C4 position of the quinolinone into the subpocket. Compound **2** has a small ethylamine substituent at the C4 position that displaces one water from the network and has only a 2-fold improvement in potency compared to compound **1**. The addition of a pyrimidine ring to give compound **3** displaced a further water molecule from the network and gave a >10-fold increase in potency. The final water was displaced by a second methyl group added to give compound **4**, improving the potency over 50-fold compared to compound **1**. An overview of the crystal structures of compounds **1**–**4** can be found in Figure S1, highlighting the key interactions with the protein and water molecules.

**Table 1:**
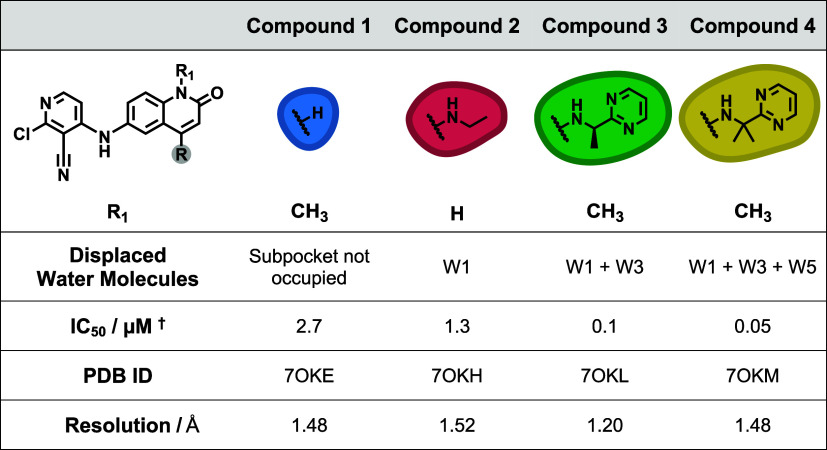
Four Compounds Selected from Lloyd et al.^11^ that Sequentially Displace up to Three Water Molecules from a Network within a Subpocket of the BCL6 Binding Site

† The IC_50_ values were calculated using TR-FRET.

We used GCMC simulations to predict both the location of the water molecules and their stability as a network for each of the four compounds in Table 1. The stability is indicated by the binding free energy of the water network, which represents the free energy change of transferring the water molecules from bulk solvent to the subpocket within the protein. By comparing the stability of the water networks in the presence of different compounds, the contribution from solvent effects to the potency changes can be predicted. For example, if a structural modification to a ligand destabilizes the water network, this could lead to a negative impact on potency.^16^ However, if the structural change introduces new interactions between the ligand and the protein or remaining water molecules, this can counteract the water network destabilization and lead to an overall improvement in potency. The effect of these new interactions on the binding affinity can be predicted using alchemical free energy calculations,^25^ which calculate the free energy of alchemically transforming one compound into another using nonphysical intermediate states. In this work, they were performed in the presence and absence of water molecules within the subpocket of interest, while leaving the rest of the protein solvated, allowing the solvent effects to be isolated.

Ultimately, GCMC and alchemical free energy calculations can be combined to design a free energy cycle, allowing us to dissect and rationalize the relative contributions to protein–ligand binding. Furthermore, this allows the calculation of cycle closure error, where the sum of the relative free energy terms around the cycle should theoretically equal zero for consistent and well-converged simulations. This methodology has been previously used to rationalize the 10-fold potency difference between two enantiomers of DPP1 inhibitors,^10^ where the *S*-enantiomer of a hydroxyl group was found to stabilize a network of two water molecules 2.9 kcal mol^–1^ more than the *R*-enantiomer. This work looks to demonstrate how beneficial these methods are at guiding the drug design process when dealing with a large and complex water network.

## Results and Discussion

We started the project by using GCMC simulations to predict the locations of the water network in the presence of the compounds from Table 1 and the positions clustered over the simulation are shown in Figure 2. We were pleased to see that GCMC was able to predict 94% of the crystallographic sites in the subpocket within 1.5 Å, considering water sites with an occupancy of 30% or higher during the simulation under physiological conditions (B_equil_).

**Figure 2:**
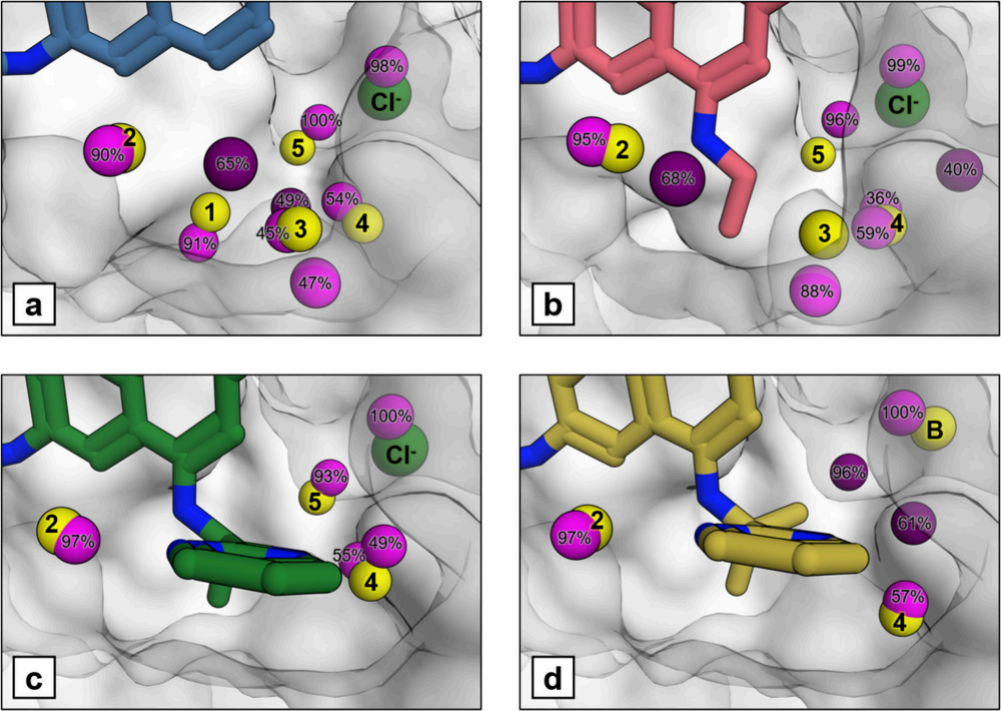
Clustered sites for oxygen positions from a GCMC simulation under physiological conditions (B_equil_) are shown as purple spheres for (a) compound **1**, (b) compound **2**, (c) compound **3** and (d) compound **4**. These are labeled with their occupancy over the simulation and clusters with an occupancy below 30% are not displayed. The water molecule and chloride ion positions from the X-ray crystal structure (PDB ID: (a) 7OKE, (b) 7OKH, (c) 7OKL, (d) 7OKM) are shown as yellow and green spheres respectively. Clustered sites that are more than 1.5 Å away from a crystallographic position are shown in a darker shade.

The simulations revealed an occupancy of 7 water molecules in the subpocket for compound **1**, predicting two extra water sites compared to the X-ray crystal structure with a conserved network of 5 water molecules. One of the additional sites is occupied by a chloride ion in the crystal structures of compounds **1**–**3**, but corresponds to a water molecule in the crystal structure of compound **4**, labeled as site B in Figure 2d. This provides further confidence in the GCMC predictions. The other additional site has low corresponding electron density in the crystal structure and we attributed this to the fact that GCMC can identify the more disordered or solvent-exposed water sites that would have low electron density in crystal structures. In general, computational simulations find more water sites than seen in experimental X-ray crystal structures,^15,26^ which represent an averaged snapshot of the protein–ligand system.^27^

We next compared the predictions of the water network for compounds **1** to **4** in Figure 2. GCMC predicted that the newly introduced amine substituent of compound **2** displaced W1, and the significantly larger pyrimidine substituent of compound **3** not only displaced W1 but also W3, matching the experimental observations from the respective X-ray crystal structures. As shown in Figure S2a, only one GCMC simulation of compound **1** out of three repeats had a site corresponding to W4, preferring to occupy sites shifting toward W1. For the other compounds, the W4 site is split over several sites to make better interactions with neighboring water molecules, such as W3 and W5 for compound **2**, or with the pyrimidine substituent for compounds **3** and **4**. This suggests that this site is less stabilized by the protein and more mobile than other water molecules in the network.

By inspecting the crystal structures of all the compounds, we noticed a difference in the binding site of compound **4**, where His116 had a different orientation compared to the other compounds, shown in Figure S2d. As the His116 residue was pointing into the subpocket, the use of this crystal structure led to displacement of water molecules in the right side of the subpocket by the protein during the simulation, predicting occupancy for only one water molecule at the W2 site as shown in Figure S2e. Therefore, we used the protein structure from the crystal structure of compound **3** for the simulations of compound **4**, allowing the comparison of the GCMC predictions within a similar protein environment. This also reflects the scenario of a prospective application where different analogues of a compound would be simulated in the same binding site. Interestingly, the clustered water positions from the simulation of compound **4** in Figure 2d show a site corresponding to W5 which was not present in the X-ray structure of compound **4**. The comparison to the water positions from the crystal structure of compound **3** can be found in Figure S2f. This fits with the previous SZMAP analysis on the crystal structure of compound **3** that predicted W5 to be strongly bound to the protein and difficult to displace.^11^ The displacement of W5 could result from a change in the protein structure, such as His116 side chain orientation, that requires sampling from other computational techniques, such as molecular dynamics.^28^

Having demonstrated that the predicted positions of the water network were consistent with the experimental crystal structures, we next used GCMC to calculate the binding free energy of the water networks. To do that, GCMC simulations at different chemical potentials (B values) were used to predict the stability of the water network in the presence of each compound, as shown in Figure 3. As the chemical potential decreases, water molecules are removed from the binding site until the occupancy reaches zero, giving the titration plots in Figure 3a. The individual results from three sets of GCMC simulations that can be found in Figure S3. Using the area underneath the titration curve for the unsubstituted compound **1**, the binding free energy of the water network (Δ*G*_1,W1–7 bind_) is predicted to be −14.5 ± 0.6 kcal mol^–1^, as shown by the blue line in Figure 3b. Compound **2** displaces one water molecule from the network with the addition of the amine substituent and the occupancy of the subpocket decreases from 7 to 6 water molecules. Comparing the blue and pink lines in Figure 3b, the GCMC simulations indicate that the remaining water network has been destabilized by around 6 kcal mol^–1^. This huge change in the stability of the water network would be unfavorable and would negatively impact the overall binding potency of compound **2**.

**Figure 3:**
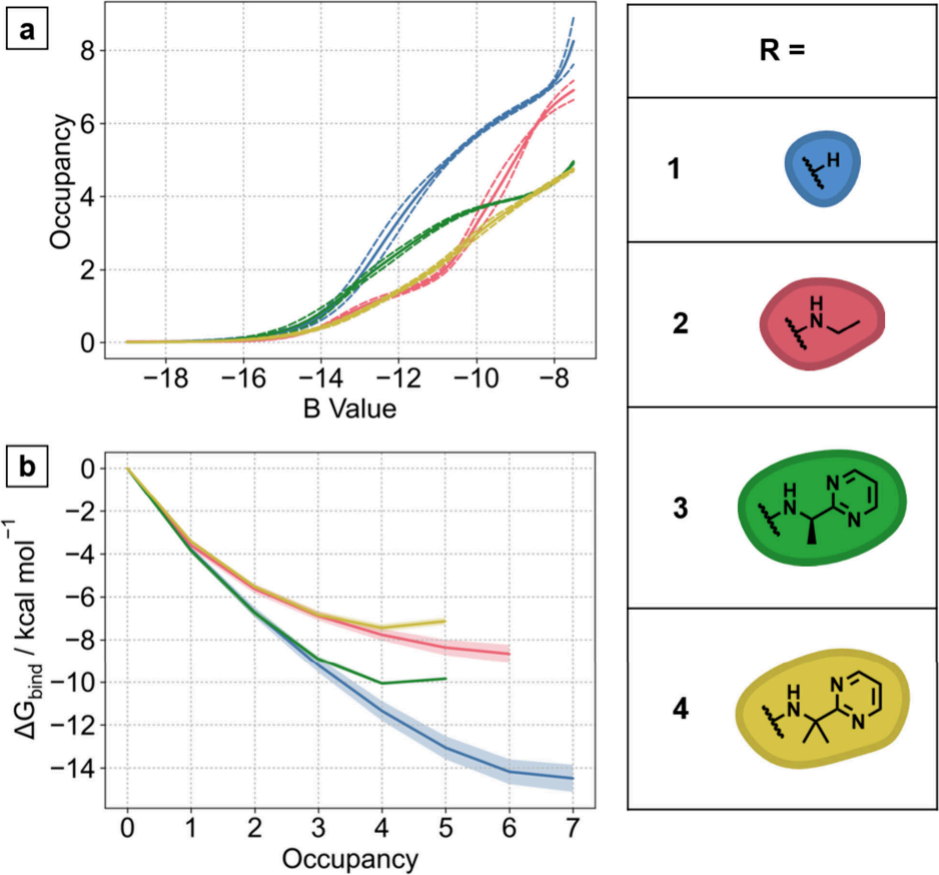
(a) GCMC simulations of each compound are performed at different chemical potentials (B values) and the occupancy of the subpocket is calculated at each value. (b) Binding free energy of the water molecules in the subpocket compared to bulk solvent can be calculated through the Grand Canonical Integration method,^19^ using the integration of the results shown in (a).

The amine substituent in compound **2** engages in additional interactions with the protein compared to compound **1**, making a hydrogen bond to the backbone carbonyl of Ala52 and lipophilic contacts with Val18 shown in Figure S1b. Alchemical free energy calculations were used to predict the contribution of these interactions made by the new substituent toward the binding affinity of compound **2**. Surprisingly, these calculations showed that growing the compound by an ethylamine group and extending into the subpocket is predicted to be nearly 6 kcal mol^–1^ more favorable in the absence of water molecules (Δ*G*_**2**→**1**, no_W1–6_) than in the solvated subpocket (Δ*G*_**2**→**1**,W1–6_). This indicates that the addition of the amine substituent has a destabilizing effect on the water network that negatively impacts potency. Figure 4, with an absolute cycle closure error of 0.3 kcal mol^–1^, indicates that the alchemical free energy results are consistent with the GCMC simulations and well-converged. The cycle suggests that the introduction of the ethylamine group has two opposing effects on potency: it engages in additional interactions that have a positive effect on binding affinity, but the destabilization of the water network has a negative effect. These opposite effects nearly cancel each other out, rationalizing why overall the potency only has a 2-fold improvement with IC_50_ values of 2.7 and 1.3 μM for compound **1** and **2** respectively.

**Figure 4:**
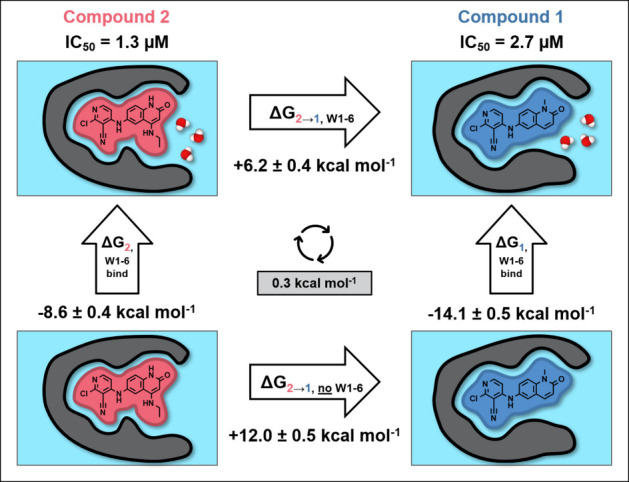
Free energy cycle for the transformation of compound **2** to **1**. The horizontal arrows indicate the free energy for the alchemical perturbation of the compounds calculated in the presence (top) and absence (bottom) of water in the subpocket (W1–6), not including the seventh water molecule that was displaced by the added substituent (W1 in Figure 1). The vertical arrows indicate the binding free energy of W1–6 to the protein–ligand system calculated by GCMC in the presence of compound **1** (right) and compound **2** (left). The cycle closure error is shown in the gray box in the center.

Interestingly, the lack of potency increase was rationalized in the original paper^11^ by the suboptimal geometry of the new hydrogen bond between the new amine substituent and Ala52 and a shift in the scaffold that could have impacted other interactions made by the core. However, our results suggest that the modest gain of compound **2** is due to the destabilizing effect on the water network.

The realization that the conversion from compound **1** to **2** destabilizes the water network has an important implication: the remaining water molecules are easier to displace with subsequent substitutions. While the potency gain for compound **2** was minimal, it laid the basis for larger potency gains through additional modifications as replacing further water molecules would incur a smaller penalty. This would have been valuable insight if available during the project, supporting design around further exploration of this subpocket, despite discouraging IC_50_ values.

Having rationalized the potency trend for the transition for compound **1** to compound **2**, we next used GCMC and alchemical free energy calculations to look at the change from compound **2** to compound **3**. Previous SZMAP analysis^11^ of compound **2** pointed to replacing the remaining water molecules with a polar substituent and various amide substituents were added to the ethyl group. Following a change to heteroaromatic isosteres to improve passive permeability, compound **3** with a pyrimidine substituent was prepared and showed over 10-fold increase in potency. Notably, analysis of the X-ray structure revealed that the pyrimidine group displaced both W1 and W3 from the water network. Given the destabilization of the water network caused by compound **2**, it would be reasonable to expect that a larger group would further destabilize the remaining water molecules. Remarkably, the results in Figure 3b predict that the water network is stabilized by around 1.5 kcal mol^–1^, despite the displacement of W3, and this would contribute significantly to the overall gain in potency. This stabilization of water network can be rationalized by nitrogen atoms on the pyrimidine ring making new interactions with W2 and W4, following the displacement of W1 and W3. These hydrogen bond interactions can be seen in Figure S1c. The stabilizing effect of the pyrimidine substituent on the water network is also reflected in the alchemical free energy calculations shown in Figure 5. The transformation from compound **2** to **3** is more favorable in the presence of water in the subpocket (Δ*G*_3→2,W1–5_), compared to the reverse being true in the first cycle for the transformation from compound **1** to **2** in Figure 4.

**Figure 5:**
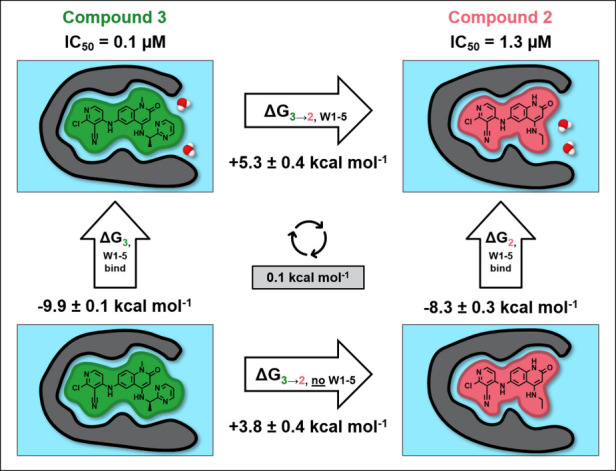
Free energy cycle for the transformation of compound **3** to **2**. The horizontal arrows indicate the free energy for the alchemical perturbation of the compounds calculated in the presence (top) and absence (bottom) of water in the subpocket (W1–5), not including the sixth water molecule that was displaced (W3 in Figure 1). The vertical arrows indicate the binding free energy of W1–5 to the protein–ligand system calculated by GCMC in the presence of compound **2** (right) and compound **3** (left). The cycle closure error is shown in the gray box in the center.

Compound **3** makes the same polar contacts with the protein as compound **2** and displaces a further water molecule from the network, as shown in Figure S1c. Nonetheless, the pyrimidine replaces the hydrogen bond interactions made by that water within the network and the cycle in Figure 5 justifies the over 10-fold improvement in potency seen for the (R)-enantiomer. This pyrimidine analogue was described as a ‘significant breakthrough’ in the project,^11^ due to the improvement of both potency and permeability that allowed progression to the cellular assay. The cycle closure error of 0.1 kcal mol^–1^ indicates that the computational predictions for the energetics of both the water network and ligands binding to the protein are consistent and reliable. This demonstrates the capability of these methods to handle large changes in the ligand structure and subsequent effects on the water network.

Finally, we used GCMC and alchemical free energy calculations to study the addition of a methyl group to the chiral carbon of the ethyl group to give compound **4**. This second methyl group was introduced to further improve the permeability of compound **3** by increasing the lipophilicity. Previous SZMAP analysis^11^ of compound **3** indicated that displacing W5 with a methyl group would not be favorable as it was predicted to have a ΔΔ*G* value of −6 kcal mol^–1^. However, compound **4** displaced W5 according to the X-ray crystal structure, as shown in Figure S1d, and had a 2-fold gain in potency, contrary to the SZMAP results.

The stability of the water network is predicted by GCMC to decrease by around 2 kcal mol^–1^ with the addition of the second methyl group, shown by the green and yellow lines in Figure 3b. Although the displacement of W5 is not observed computationally, GCMC predicts the water network to be destabilized by the close contact of the methyl group.

Previous conformational analysis^11^ indicated that the additional methyl group stabilized the bioactive conformation, giving an explanation for the increase in potency. This fits with the alchemical free energy calculation in Figure 6 where the addition of the second methyl group to compound **3** results in a favorable free energy change when there is no water molecules present in the subpocket (ΔG_4→3, no_W1–5_). However, the benefit of the prearrangement is compensated by the destabilization of the water network, rationalizing the small 2-fold improvement in potency between compounds **3** and **4**, similar to the first cycle in Figure 4. The cycle closure error of 0.9 kcal mol^–1^ is the largest of the three cycles in Figures 4–6, likely due to the use of the same protein crystal structure for both compounds, but the error is within 1.0 kcal mol^–1^, indicating that the simulations are still accurate.

**Figure 6:**
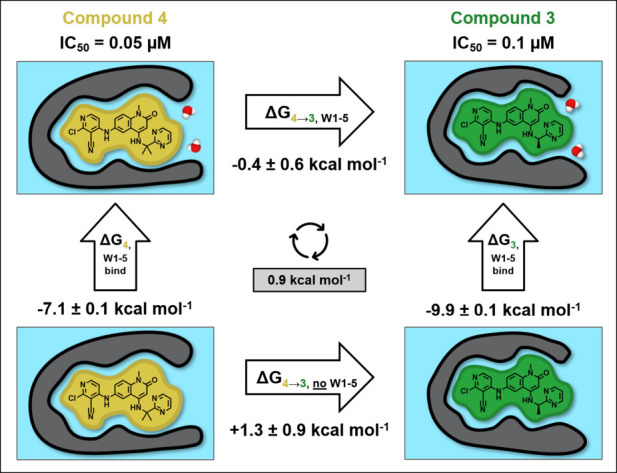
Free energy cycle for the transformation of compound **4** to **3**. The horizontal arrows indicate the free energy for the alchemical perturbation of the compounds calculated in the presence (top) and absence (bottom) of water in the subpocket (W1–5), not including the sixth water molecule that was displaced (W3 in Figure 1). The vertical arrows indicate the binding free energy of W1–5 to the protein–ligand system calculated by GCMC in the presence of compound **3** (right) and compound **4** (left). The cycle closure error is shown in the gray box in the center.

The 3D-RISM method was also used to analyze the water network for compounds **1**–**4** and the results are shown in Figure S4. In general, the majority of the 3D-RISM predicted water sites have unstable free energy values, indicating the stabilizing interactions between the water molecules seem to not be calculated by the method. For example, Figure S4a shows the 3D-RISM predictions for compound **1**, where W1 is predicted to be unstable with a free energy of +1.85 kcal mol^–1^. This prediction would indicate that the water is suitable for displacement to provide a boost in potency, which does not fit with the observed potency change when it is displaced by the amine group of compound **2**. Additionally, the stabilizing effect from the pyrimidine ring of the ligand does not seem to be captured, with predicted free energies for W2 and W4 becoming more positive for compounds **3** and **4** in Figure S4c and d respectively, compared to compound **2** in Figure S4b. By comparison to the faster 3D-RISM method in Figure S4 and the SZMAP analysis used by Lloyd et al.,^11^ the benefits of using computational simulations for solvent analysis can be seen, with the free energy cycles in Figures 4–6 providing clear rationale for the trend in IC_50_ values for compounds studied.

Alchemical free energy calculations are often used in drug discovery to calculate the relative potency changes between compounds sharing a common scaffold, termed Relative Binding Free Energy (RBFE) calculations,^29^ as calculating the absolute binding free energy of a compound is computationally more expensive. By running additional simulations with perturbations in bulk solvent (Δ*G*_solvent_) and using the bound values from Figures 4–6, the relative binding free energies (ΔΔ*G*_bind_) for the compound studied can be calculated, which are found in Table S1. However, neither the bound values in presence nor in absence of the water network give ΔΔ*G*_bind_ values that correlate with the experimental values calculated using the IC_50_ values. The major limitation of this approach is that the number of water molecules in the subpocket has to remain constant and the water molecule displacement is not captured.

This work shows the power of GCMC and alchemical free energy calculations to quantify differences in the stability of a water network in the presence of different compounds, but are not the appropriate tools for predicting binding affinity. Overall, these results support the use of GCMC sampling in free energy perturbation protocols^30,31^ to capture the perturbation of water networks.

## Conclusion

We used the combination of two computational methods to rationalize the structure–activity relationship of compounds that displaced water molecules from a network in the BCL6 binding site.^11^ We demonstrated here how alchemical free energy calculations can be used to complement the results from Grand Canonical Monte Carlo (GCMC) simulations,^10,21,22^ which can predict the thermodynamics of water networks while accounting for the cooperative interactions between the water molecules.

We focused on four BCL6 inhibitors, each with a high-resolution crystal structure, that sequentially displace water molecules from a network and achieve a 50-fold improvement in potency. This provided a perfect case study to showcase the power of GCMC simulations. For example, we found that GCMC simulations were able to reproduce the experimentally observed water sites from the crystal structures with a sensitivity of 94%, even when starting from a protein structure crystallized with a different compound, indicating that these methods can be utilized early in the lead optimization stage.

GCMC and alchemical free energy calculations were used to quantify the individual contributions from the changes in the solvent and new interactions with the protein respectively. This allowed the deconvolution of potency changes across the four BCL6 inhibitors. For instance, compounds **2** and **4** demonstrated how the balancing of these two factors can limit the improvement of potency. The new substituents for these compounds destabilized the water network, causing a detrimental impact on potency that canceled out the benefits from the additional protein interactions. In contrast, compound **3** showed stabilization of the water network by over 1.5 kcal mol^–1^, rationalizing the 10-fold increase in potency. These computational methods gave precise results for this case study, consistent with each other, as demonstrated by cycle closure errors below 1 kcal mol^–1^ for all compounds studied.

As with many drug discovery efforts, these compounds were taken from a project that took years of work and optimally perturbing the water network took several iterations of synthesis and testing. We believe that insights from GCMC calculations would have been impactful to accelerate the drug design process, highlighting the more favorable groups to occupy the subpocket and resulting in fewer rounds of synthesis. The time scale and computational expense of these simulations are reasonable, with the ability to perform GCMC calculations overnight, especially if the results are guiding compound design where the synthesis is challenging and time-consuming. By using BCL6 as a retrospective case study, we hope to encourage the use of GCMC more widely in prospective applications, guiding drug design when working with large water networks in the binding site.

## Methods

### Protein–Ligand Complex Preparation

The crystal structure was loaded from the RCSB PDB database (see Table 1 for PDB IDs) as the biological assembly to give the protein dimer and prepared using Molecular Operating Environment.^32^ Structural issues were corrected, and Protonate3D^33^ was used to add hydrogens and assign protonation states at pH 7.

### General Simulation Details

The two protein chains and one ligand structure were isolated for simulation. Water molecules from the crystal structure and compounds used for crystallography, including the WVIP peptide, were not included in the simulations. Protein residues farther than 20 Å from the ligand were removed to reduce the computational resources required. During the simulation, residues over 16 Å from the ligand had a fixed backbone with only side chain movement. The residues within 16 Å of the ligand had a flexible side chain and backbone.

The protein residues and ligands were modeled using the AMBER ff14SB^34^ and GAFF16 ^35^ force fields respectively, and solvated in a sphere of TIP4P^36^ water molecules with a radius of 30 Å, fixed with a half-harmonic potential with force constant of 1.5 kcal mol^–1^ Å^–2^. AM1-BCC partial charges^37,38^ were assigned to the ligands.

All simulations were performed using ProtoMS 3.4.^39^ They were run at 298 K and used a cutoff of 10 Å for the nonbonded intermolecular interactions, with a switching function applied to the last 0.5 Å.

Each GCMC simulation and alchemical perturbation was run on an Intel Xeon Platinum 8260 processor, with one B value and one lambda per core, respectively. The GCMC simulations took approximately 4 h per compound and the alchemical perturbations took approximately 72 h per transformation.

### GCMC Simulations

The GCMC region was defined as a bounding box around the oxygen atoms of the water network from the crystal structure of compound **1** (PDB ID: 7OKE) with 1 Å padding. Any water molecules in the GCMC region were removed before the simulation. The simulations were run in the μVT ensemble, allowing the number of water molecules in the system to vary while keeping the chemical potential of bulk water, volume and temperature fixed throughout. The chemical potential is represented by a B value.^24^ GCMC simulations were performed at 24 B values from −19.0 to −7.5 at 0.5 intervals, with replica exchange^22^ between adjacent B values attempted every 100,000 moves. Each simulation was equilibrated for 5 million (5M) moves where only the solvent within the GCMC region was sampled. A further equilibration of 5 M moves also sampled the protein, ligand and bulk solvent. 40 M production moves were performed, with coordinates of the system saved every 100,000 moves. Three independent sets of 24 simulations were performed for each compound.

The Grand Canonical Integration method^21,22^ was used to calculate the binding free energy of the water networks within the GCMC region. The average value for the three independent repeats is reported with the standard error.

The position of the oxygen atoms over the simulation at B_equil_ were clustered using average-linkage hierarchical clustering with a distance cutoff of 2.4 Å.

The accuracy of the GCMC simulation at B_equil_ was measured using the true positive rate (TPR or sensitivity) in eq 1 defined by Samways et al.:^15^

1 $$\mathrm{TPR}=\frac{\mathrm{TP}}{\mathrm{TP}+\mathrm{FN}}=\frac{\#\mathrm{correct predictions}}{\#\mathrm{crystallographic sites}}$$

where a true positive (TP) indicates a predicted water site within 1.5 Å of a crystallographic site and a false negative (FN) indicates a crystallographic site with no predicted water site.

Predicted sites with an occupancy lower than 30% were not included.

### Alchemical Free Energy Calculations

The alchemical free energy calculations^25^ involved the alchemical transformation of one ligand into another over the nonphysical coordinate λ. This was performed in the canonical ensemble using a dual topology approach, where both ligands were present in the simulation and the interactions of one ligand were gradually turned on while the interactions of the other ligand were gradually turned off as the value of λ changes from 0 to 1. This process used a softcore potential for the nonbonded energy terms, where the parameters δ and δ_c_ are used to control the soft-core Lennard-Jones and Coulomb potentials respectively. These parameters were optimized for each transformation:^25,40^ δ = 0.2 was used for all transformations, δ_c_ = 2.5 for **2** → **1** and δ_c_ = 2.0 for **3** → **2** and **4** → **3**.

To avoid a ligand drifting when it was completely turned off and had no interactions with its surroundings, the ligands were constrained together using a dummy bond between their center of geometries, and their rigid-body translations and rotations were coupled.

Each simulation used 16 equally spaced λ windows, with replica exchange swaps between adjacent λ values attempted every 100000 moves. The system was equilibrated for 5 M moves, sampling the protein, ligand and bulk solvent. This was then extended for the production stage of 40 M moves, saving the coordinates of the system every 100000 moves. The number of moves of the equilibration and production stages were doubled to 10 and 80 M respectively for the **2** → **1** and **3** → **2** due to the size of the transformations.

The free energy difference was calculated using Multistate Bennett Acceptance Ratio (MBAR).^41^ Four independent simulations were performed for each transformation, two as protein–ligand complexes with the presence and absence of water molecules in the protein subpocket in the starting coordinates and another in bulk solvent without the protein structure. The average value for the free energy differences and standard deviation are reported.

### 3D-RISM Calculations

3D-RISM calculations were performed using Molecular Operating Environment.^32^ The partial charges of the proteins and ligands were calculated using the AMBER ff19SB force field^42^ and AM1-BCC charge model.^37,38^ A 3D grid was generated around the protein–ligand complex with a minimum distance of 7 Å from the solute atoms and a grid spacing of 0.35 Å.

## Supplementary Material



## Data Availability

The crystal structures of the compounds in this work are available from the RCSB PDB database with PDB IDs listed in Table 1. All simulations were performed using ProtoMS 3.4,^35^ which is freely available at https://www.essexgroup.soton.ac.uk/ProtoMS/index.html. The starting coordinates and the random number seeds for the simulations, along with example input scripts, are provided at https://github.com/danihares/decoding-bcl6-inhibitors.

## Notes

Additional figures showing X-ray crystal structures of compounds **1**–**4** in Figure S1; GCMC clustering results from all simulation repeats in Figure S2; GCMC titration results from all simulation repeats in Figure S3; 3D-RISM results in Figure S4; RBFE results for the compounds **1**–**4** using perturbation in bulk solvent in Table S1 (PDF)

## Abbreviations

BCL6: B-Cell Lymphoma 6
GCMC: Grand Canonical Monte Carlo
MBAR: Multistate Bennett Acceptance Ratio
RBFE: Relative Binding Free Energy
TR-FRET: Time-Resolved Fluorescence Energy Transfer
