# Supplementary material for: Decoding BCL6 Inhibitors: Computational Insights into the Impact of Water Networks on Potency
Source: J Chem Inf Model. 2025 Aug 28;65(18):9557–65. doi: 10.1021/acs.jcim.5c01188 (PMC12458693; doi:10.1021/acs.jcim.5c01188)
Supplement: Supplementary file 1 [file ci5c01188_si_001.pdf]

Supporting Information

Decoding BCL6 Inhibitors: Computational Insights into the Impact of Water Networks on Potency

Authors

Daniella E. Hares[1], Andrea Scarpino[1], Michael S. Bodnarchuk[2]*, Swen Hoelder[1]*

[1] Centre for Cancer Drug Discovery, The Institute of Cancer Research, London SM2 5NG, UK

[2] Oncology R&D, AstraZeneca, Cambridge Biomedical Campus, Cambridge CB2 0AA, UK

Corresponding Authors

*Email: swen.hoelder@icr.ac.uk

*Email: michael.bodnarchuk@astrazeneca.com

Table of Contents

Figure S1: X-ray crystal structures of compounds **1-4**..2

Figure S2: GCMC clustering results for all repeats 3

Figure S3: GCMC titration results for all repeats 4

Figure S4: 3D-RISM results for compounds **1-4**...5

Table S1: RBFE results using perturbation in bulk solvent...6

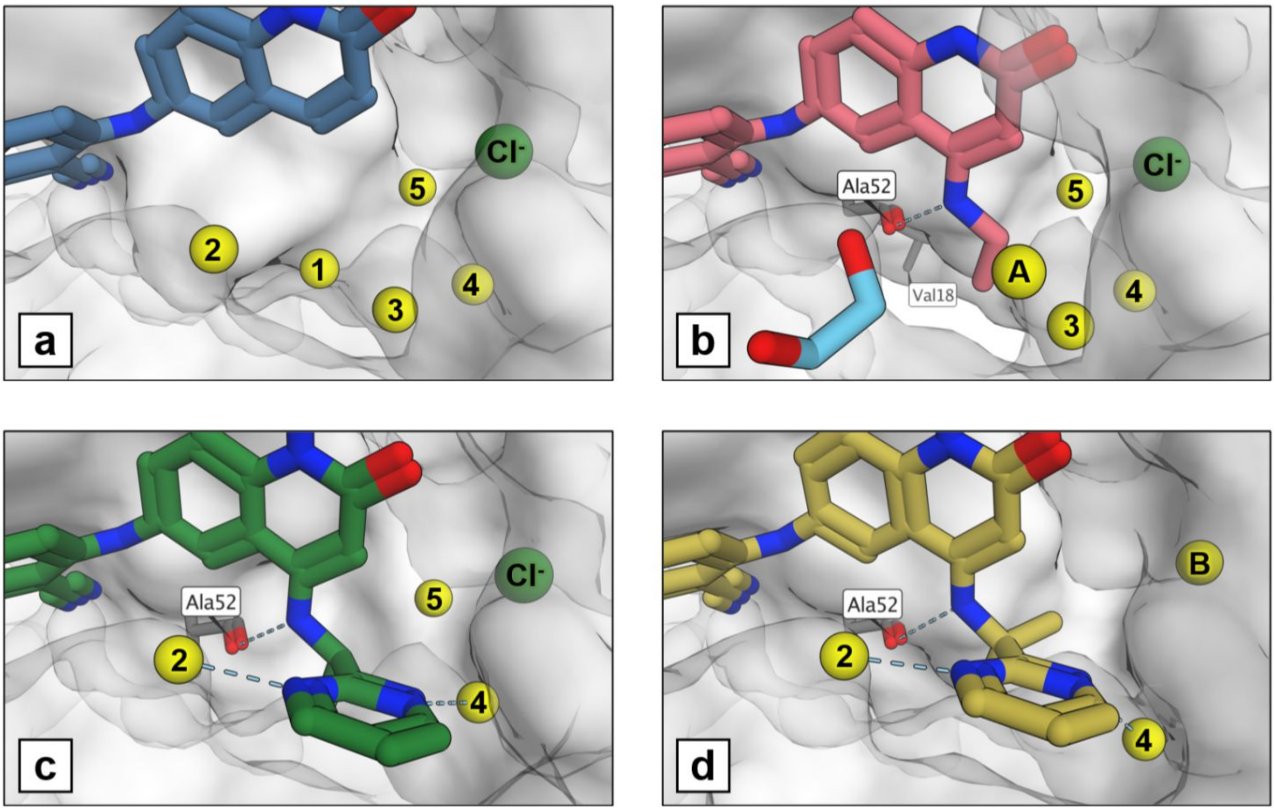

Figure S1: X-ray crystal structures of (a) compound **1**, (b) compound **2**, (c) compound **3** and (d) compound **4** (PBD ID: (a) 7OKE, (b) 7OKH, (c) 7OKL, (d) 7OKM), showing the key interactions made with the protein residues in grey. The crystallographic positions of the water molecules and chloride ions are shown as yellow and green spheres respectively. The crystal structure of compound **2** has an ethylene glycol molecule in the W2 position, shown in cyan, potentially resulting in additional water position A.

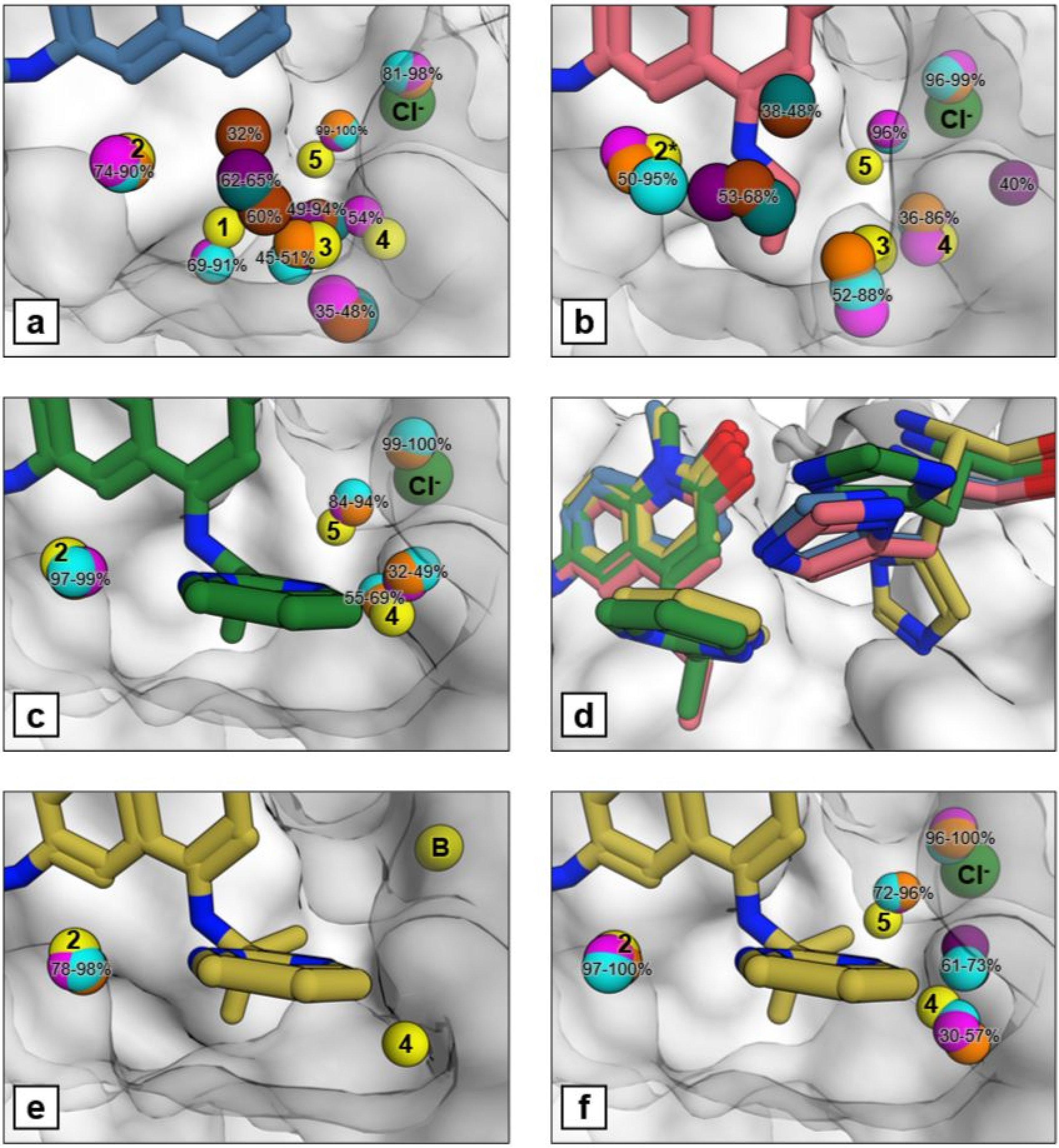

Figure S2: Clustered sites for oxygen positions from three independent GCMC simulations under physiological conditions (B_{equil}) are shown as different coloured spheres (purple, orange and cyan) for (a) compound **1**, (b) compound **2**, (c) compound **3**, (e) compound **4** simulated using the crystal structure 7OKM and (f) using the crystal structure 7OKL. These are labelled with their occupancy over the simulation and clusters with an occupancy below 30% are not displayed. The water molecule and chloride ion positions from the X-ray crystal structure (PBD ID: (a) 7OKE, (b) 7OKH, (c) 7OKL, (e) 7OKM, (f) 7OKL) are shown as yellow and green spheres respectively. Clustered sites that are more than 1.5 Å away from a crystallographic position are shown in a darker shade. *The position for W2 was occupied by an ethylene glycol molecule in the structure for compound **2** (PDB ID: 7OKH, so the W2 position from the structure of compound **1** (PDB ID: 7OKE) has been shown for clarity. (d) Crystal structures of compounds **1**-**4** showing positions of the His116 residue in binding site.

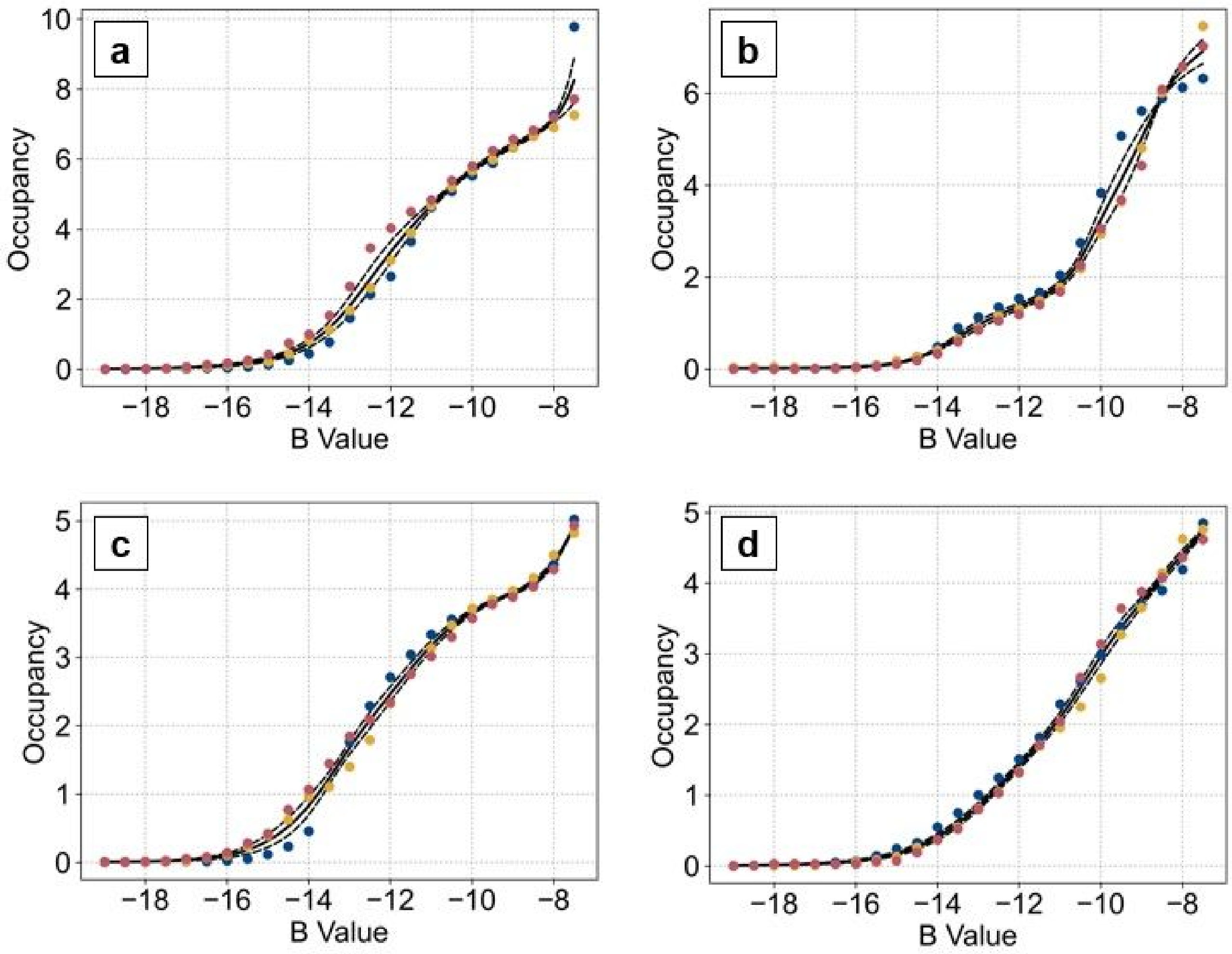

Figure S3: Titration results from three independent sets of GCMC simulations at 24 B values for (a) compound **1**, (b) compound **2**, (c) compound **3** and (d) compound **4**.

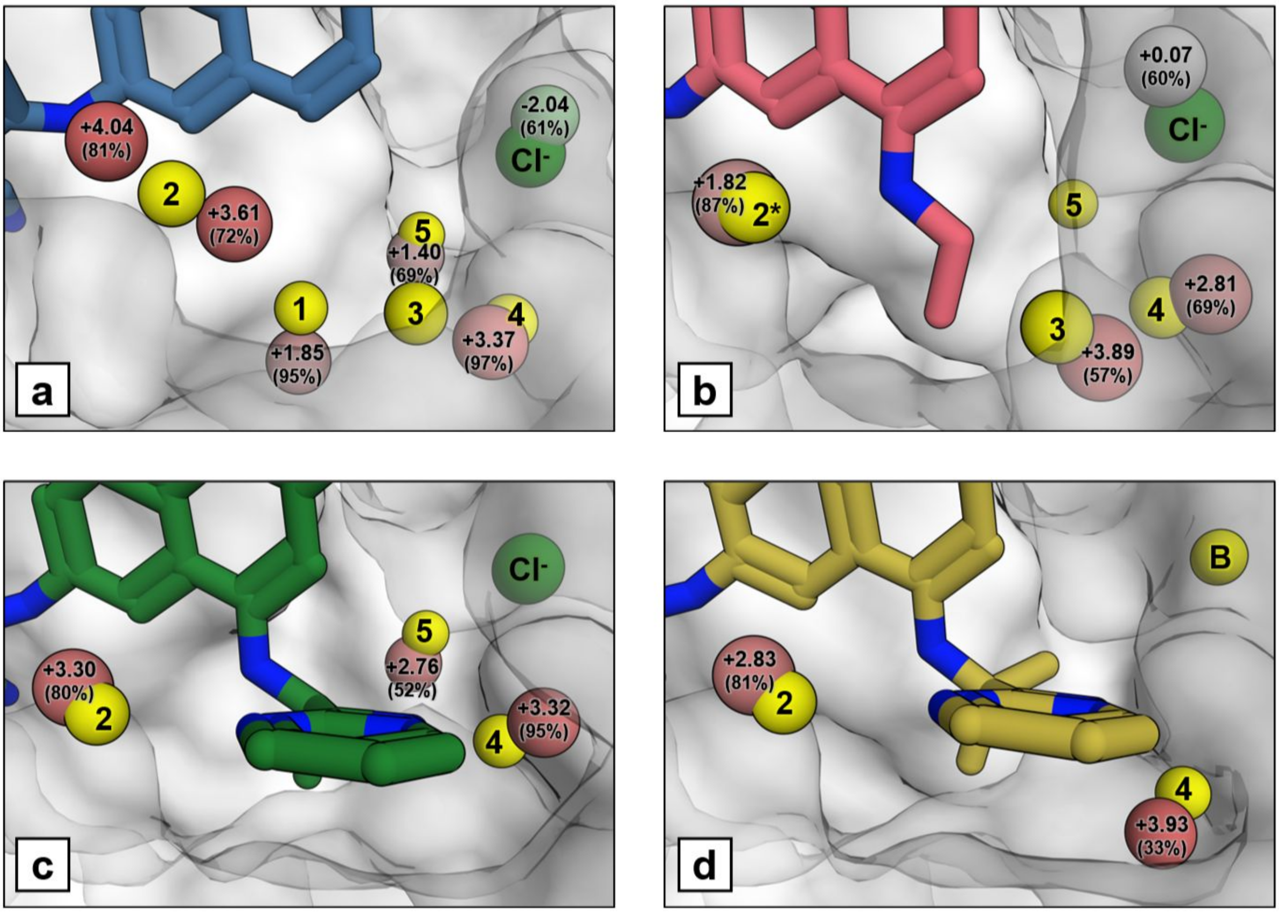

Figure S4: 3D-RISM results for (a) compound **1**, (b) compound **2**, (c) compound **3** and (d) compound **4**. The water molecule and chloride ion positions from the X-ray crystal structure (PBD ID: (a) 7OKE, (b) 7OKH, (c) 7OKL, (d) 7OKM) are shown as yellow and green spheres respectively. The predicted sites within 1.5 Å of the crystallographic positions are shown with their predicted free energy (kcal mol^{-1}) and occupancy values, and colour-coded by their free energies. *The position for W2 was occupied by an ethylene glycol molecule in the structure for compound **2** (PDB ID: 7OKH, so the W2 position from the structure of compound **1** (PDB ID: 7OKE) has been shown for clarity.

Table S1: Relative binding free energy values ($\Delta\Delta G_{bind}$) for the transformations between compound **1-4** using a perturbation in bulk solvent ($\Delta G_{solvent}$). All values are in kcal mol^{-1}.

	**$\Delta G_{bound, W}$**	**$\Delta G_{bound, no W}$**	**$\Delta G_{solvent}$**	**$\Delta\Delta G_{bind, W}$**	**$\Delta\Delta G_{bind, no W}$**	**$\Delta\Delta G_{expt}$**
**2** → **1**	+6.2 ± 0.4	+12.0 ± 0.5	+3.3 ± 0.1	+2.9 ± 0.5	+8.7 ± 0.6	+0.4
**3** → **2**	+5.3 ± 0.4	+3.8 ± 0.4	+1.3 ± 0.2	+4.0 ± 0.6	+2.5 ± 0.6	+1.5
**4** → **3**	-0.4 ± 0.6	+1.3 ± 0.9	+1.6 ± 0.3	-2.0 ± 0.9	-0.3 ± 1.2	+0.4

The relative binding free energy was calculated according to the following equation:

$$\Delta\Delta G_{bind} = \Delta G_{bound} - \Delta G_{solvent}$$

$\Delta\Delta G_{bind}$ was calculated using the ΔG_{bound} values from the perturbations when bound to the protein, both with ($\Delta G_{bound, W}$) and without ($\Delta G_{bound, no W}$) the water network in the subpocket.

$\Delta\Delta G_{expt}$ was calculated using the following equation:

$$\Delta\Delta G_{bind} = \Delta G_2 - \Delta G_1 = RT \ln\left(\frac{K_1}{K_2}\right)$$

where R is the gas constant, T is the temperature (298 K) and K is the dissociation constant which is approximated from the IC_{50} value.
